# Jugulotympanic Paraganglioma With Preoperative Embolization That Led to Facial Nerve Paralysis and Surgical Rerouting of the Nerve

**DOI:** 10.7759/cureus.41997

**Published:** 2023-07-17

**Authors:** Katerina Marini, Vasiliki Florou, James Philip Skliris, Nikolaos Marangos, Nikolaos Kamargiannis

**Affiliations:** 1 Department of Otorhinolaryngology-Head and Neck Surgery, 'G. Gennimatas' General Hospital, Thessaloniki, GRC; 2 Department of Pathology, 'G. Papanikolaou’ General Hospital, Thessaloniki, GRC; 3 Department of Otolaryngology-Head and Neck Surgery, Center of ENT and Head and Neck Surgery, Rottweil, DEU

**Keywords:** oncology, otolaryngology, surgical excision, rerouting, embolization, paraganglioma

## Abstract

Paragangliomas are mostly benign, slow-growing, hypervascular tumors originating from neural crest derivatives. Head and neck (H&N) paragangliomas represent <1% of all H&N tumors and <5% are malignant. They are mostly non-secreting tumors that originate from autonomous parasympathetic paraganglia. We present a case of right middle ear jugulotympanic paraganglioma, a subtype of H&N paragangliomas, which had been misdiagnosed as otosclerosis for about 10 years. The patient was suffering from worsening tinnitus along with hearing impairment. High clinical suspicion of jugular paraganglioma prevented us from taking a biopsy. Complete surgical excision after preoperative embolization was decided. Embolization resulted in facial nerve paralysis, however, facial nerve rerouting was performed during the complete surgical excision of the tumor. The patient remains disease-free three years postoperatively, with House-Brackmann III facial nerve paralysis.

## Introduction

Paragangliomas are usually benign and slow-growing tumors that originate from neural crest derivatives [1]. When occurring at the adrenal paraganglia, they are called pheochromocytomas [2]. They can be found from the skull base to the pelvis. They are divided into sympathetic and parasympathetic paragangliomas [2]. The most common paragangliomas of the H&N are those of the carotid body, followed by jugulotympanic paragangliomas and glomus vagale tumors, and account for 0.6% of H&N tumors and about 70% of paragangliomas, excluding pheochromocytomas [3]. It is worth mentioning that the term 'glomus tumor' is no longer used by pathologists for such tumors [3]. They are mostly non-secreting parasympathetic tumors. Most H&N paragangliomas are sporadic while familial cases represent 10-50% [4]. Jugulotympanic paragangliomas represent about 20-30% of H&N paragangliomas, while below 4% metastasize [3]. The incidence is 0.07 cases per million per year with a female predilection [5]. There is still controversy about the optimal treatment. Surgical resection with or without preoperative embolization, radiation therapy, and observation without surgery are the treatments of choice [1,6]. We present a case of right middle ear jugulotympanic paraganglioma, which had been considered otosclerosis for 10 years, and after the correct diagnosis was treated with embolization followed by surgical excision with facial nerve rerouting.

## Case presentation

A 53-year-old female patient presented to our Ear Nose and Throat outpatient department complaining of a 10-year hearing impairment of the right ear. She also mentioned pulsatile tinnitus and ear fullness, progressively worsening through these 10 years. She had been primarily diagnosed with otosclerosis in the past and had been treated conservatively with monitoring in another medical setting. The patient’s medical history was otherwise normal.

**Figure 1:**
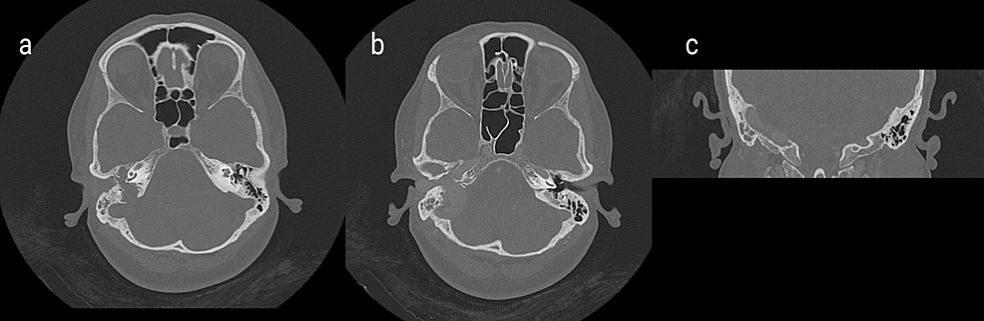
Contrast-enhanced computed tomography (CT) findings A heterogeneous soft tissue mass about 2.5x2x4 cm. (a, b) axial CT showing eroded right jugular foramen and expanding through the floor of the middle ear to the right external auditory meatus. In close proximity to the right sigmoid sinus and internal jugular vein, (c) coronal CT: intracranial extension of the lesion.

A thorough physical examination was performed. Otomicroscopy revealed a bluish mass partially covered by the epidermis, which was occupying the right external auditory meatus. The tympanic membrane could not be visualized. Neck palpation was unremarkable. No cranial nerve deficits were observed. Both Rinne and Weber tests were indicative of conductive hearing loss on the affected side. Pure-tone audiometry confirmed a right ear conductive hearing loss (tympanometry was not performed, as the auditory meatus was blocked by the mass). The patient underwent contrast-enhanced petrous bone computed tomography (CT) and magnetic resonance imaging (MRI). The imaging tests revealed an enhancing heterogeneous soft tissue lesion filling the right jugular foramen, which was depicted as eroded and dilated. The mass was expanding, through the jugular foramen, in the middle ear via its floor, occupying the ossicles, and toward the right external auditory canal. It was also adjoining the sigmoid sinus and the proximal internal jugular vein, presenting intracranial extension with pressure to the right cerebellar hemisphere. Dimensions were approximately 2.5x2x4 cm (Figures 1a, 1b, 1c). The diagnosis of glomus jugulare, classified as Di2 according to the Fisch Classification System, was confirmed.

**Figure 2:**
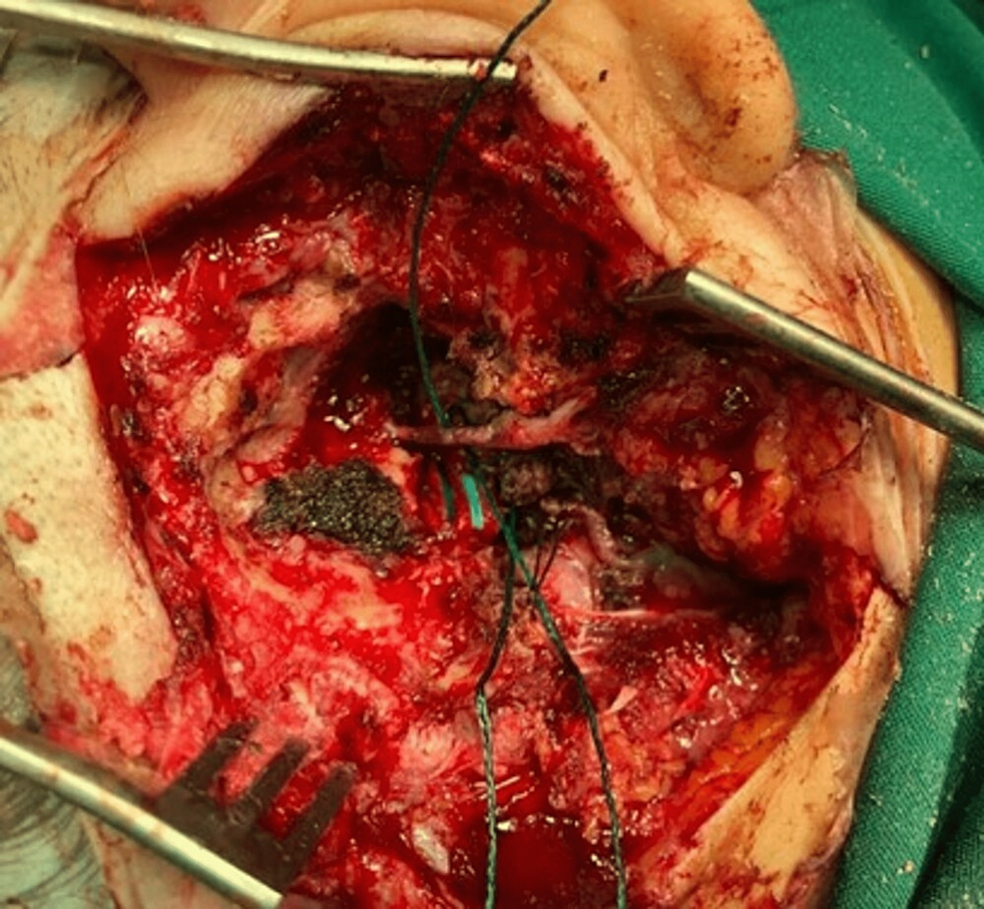
Intraoperative image: the long anterior rerouting technique for the facial nerve

The appropriate treatment for the patient was decided to be surgical removal after selective preoperative embolization. After embolization, the patient developed facial nerve paralysis (House Brackmann III) of her right face. Radical mastoidectomy under general anesthesia was performed, with complete tumor resection and facial nerve rerouting. In this procedure, the temporal part of the facial nerve and the middle ear content prevents the wide exposure of the jugular foramen area. Subsequently, to widely expose the jugular foramen and the apex of the temporal bone, the middle ear contents must be removed, and the facial nerve must be rerouted. Then, the facial nerve is mobilized along its entire mastoid and tympanic course up to the geniculate ganglion (long anterior rerouting technique). This technique is indicated for paragangliomas (Figure 2).

The cavity was filled with fat tissue, along with external auditory meatus obliteration.

**Figure 3:**
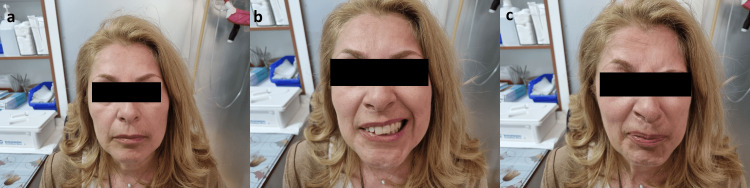
a, b, and c: Patient's three-year follow-up showing facial nerve paralysis (House Brackmann III) with adequate closure of the right eye

Following the operation, the surgical specimen was sent for histopathological examination, which confirmed the diagnosis. Round neoplastic cells with smooth nuclear membranes and abundant eosinophilic cytoplasm forming solid nests (zellballen) and trabeculae were described. Fibrovascular stroma and fibrotic areas, along with embolization-related alterations were also noticed. Paraganglioma cells were diffusely positive to synaptophysin, focally positive to chromogranin A, and positive to S-100 immunohistochemical stains. The proliferation index (Ki-67) was low at 1-2%. During the postoperative period, the patient was disease free, with House Brackmann III facial nerve paralysis. At the three-year follow-up a few weeks ago, she remains disease free with facial nerve paralysis and adequate closure of the eye (Figures 3a, 3b, 3c).

## Discussion

Paragangliomas are rare, mostly benign, slow-growing, catecholamine-secreting, hypervascular neuroendocrine tumors. They occur during neural crest migration and they are divided into sympathetic and parasympathetic tumors [4]. Their name reflects their location. The intra-adrenal paragangliomas, pheochromocytomas, secrete epinephrine, as opposed to Η&Ν ones that may secrete norepinephrine. The latter arise from parasympathetic paraganglia of the autonomous neuroendocrine system and usually do not secrete catecholamines (less than 5% are secreting and symptomatic), as opposed to adrenal paraganglia which are highly active [2]. They have also been described in the orbit, nasal cavity, paranasal sinuses, larynx, trachea, aortic body, and mediastinum [1]. Head and neck paragangliomas often cause mild and “silent” neural deficits, such as speech and swallowing symptoms in about 10% of patients [1,6].

Paragangliomas represent mostly solitary tumors but also multiple lesions have been described in 11-22% of cases [1]. H&N paragangliomas are hereditary at about 10-50% and are associated with neurofibromatosis type I, Von Hippel Lindau syndrome, Carney-Stratakis dyad, and multiple neuroendocrine neoplasia type 2A and 2B [4,6]. They include the carotid body (at the carotid bifurcation) comprising 50-60% of cases, followed by the glomus jugulare (from the jugular bulb within jugular foramen-obsolete term, jugulotympanic paraganglioma-new term), glomus tympanicum (from Arnold’s or Jacobson’s nerves within the middle ear - obsolete term, jugulotympanic - new term), and glomus vagale (from the inferior vagal ganglion - obsolete term, vagal paraganglioma - new term) tumors [3]. Despite being benign, a small percentage may be malignant or metastasize, with rates at about 6% for carotid bifurcation paragangliomas, 5% for glomus jugulare, and 17% for vagal paragangliomas [1,4]. Concerning jugulotympanic paraganglioma, it often appears to be a sporadic unilateral lesion, in the fifth to sixth decade of life, with a female predilection. It may involve cranial nerves IX-XII, causing paralysis of the affected nerves [5]. The most common symptoms are pulsatile tinnitus, ear fullness, hearing loss, or visible cervical mass [7]. When they secrete norepinephrine, paragangliomas cause also fight-or-flight symptoms such as flashing, fatigue, headache, palpitations, anxiety, etc. [3]. CT, MRI, as well as MRI angiography, are of paramount importance for diagnosis, due to the tumor’s high vascularity. Blood supply is most commonly provided by the ascending pharyngeal artery [1,4]. Urine vanillylmandelic acid levels are also tested in secreting tumors [4].

Regarding treatment, although surgical excision is recommended for large tumors, primary radiotherapy, stereotactic radiosurgery, and radiologic surveillance are also considered effective treatments. The choice depends on the patient’s health and comorbidities, tumor size and location, as well as the biological activity of the tumor [4,6]. Preoperative selective embolization followed by complete surgical excision is the safest method for jugular paragangliomas and has the lowest mortality rates [1,7]. Embolization is performed 24-48 hours prior to surgery and though it is a quite safe procedure, it could result in severe complications [4]. Thus, it requires attention and anatomic knowledge of potential dangerous anastomoses between the tumor and the important vessels [8]. For residual paragangliomas, irradiation with a Gamma Knife® is the gold standard [1]. Surgical excision is considered a demanding procedure for jugular paragangliomas due to the tumors’ high vascularity and proximity to vital structures [9,10].

## Conclusions

Otolaryngologists should always include jugulotympanic paragangliomas in the differential diagnosis of tinnitus, hearing loss, and/or ear fullness. A misleading diagnosis of otosclerosis for conductive hearing loss could lead to negligence of the underlying cause. Imaging is of paramount importance for pulsatile tinnitus or unilateral symptoms while biopsy should be avoided when there is high clinical suspicion for paragangliomas. Treatment remains challenging, as adjacent structures may be affected (e.g., the facial nerve).
